# Lipoprotein(a) Drives Platelet Hyperactivity Through Oxidized Phospholipids-CD36

**DOI:** 10.1161/CIRCRESAHA.126.329174

**Published:** 2026-09-21

**Authors:** Emily Horner, Lih T. Cheah, Thomas Palmer-Dench, Jawad S. Khalil, Mary McKay, Fraser L. Macrae, Mark T. Kearney, Amanda J. Unsworth, Khalid M. Naseem

**Affiliations:** Leeds Institute of Cardiovascular and Metabolic Medicine (LICAMM), University of Leeds, United Kingdom (E.H., L.T.C., T.P.-D., J.S.K., M.M., F.L.M., M.T.K., A.J.U., K.M.N.).

**Keywords:** blood platelets, extracellular matrix proteins, lipoprotein(a), phospholipids, src-family kinases

**Meet the First Author, see p e000772**

Lipoprotein(a) (Lp[a]) is reported to be the preferential carrier of oxidized phospholipids (oxPLs) in circulation,^1^ and elevated circulating oxPLs promote a prothrombotic state characterized by platelet hyperactivity.^2^ We observed plasma Lp(a) concentrations positively correlated with basal platelet activation in patients with coronary artery disease (r=0.5861; *P*=1.5×10^−2^; Figure [Ai]), whereas isolated Lp(a)^3^ induced primary aggregation of washed platelets (24.8%±9.7%; Figure [Aii]). We found that plasma lipid peroxidation of isolated Lp(a) (12.7±9.9 nmol/mg protein) was markedly higher than that of both native low-density lipoprotein (2.5±2.4 nmol/mg protein) and in vitro generated oxidized low-density lipoprotein (4.7±3.3 nmol/mg protein; Figure [Aiii]). Given this pronounced overall peroxidation burden, we investigated whether Lp(a) functions as a prothrombotic vehicle through its oxPL content. Lp(a) (100 µg/mL) potentiated aggregation induced by subthreshold concentrations of platelet agonists (Figure [Bi] and [Bii]). Aggregation in response to the GPVI (glycoprotein VI) agonist convulxin (12.5 ng/mL) increased from 13.2±7.6% to 56.2±22.1% (*P*=9.1×10^−6^; Figure [Bi]), whereas the thromboxane A_2_ mimetic U46619 (0.1 µg/mL) increased from 6.9±4.2% to 63.1±25.4% (*P*=9.0×10^−7^; Figure [Bii]). Critically, this Lp(a)-driven potentiation was abolished by the oxPL-neutralizing monoclonal antibody E06 (2:1 molar ratio; Figure [Bi] and [Bii]),^1^ implicating phosphocholine-containing oxPLs as the principal mediator of this response. CD36 (cluster of differentiation 36) is a high-affinity oxPL receptor on the platelet surface,^2,4^ and pharmacological blockade with the inhibitor SSO (sulfo-N-succinimidyl oleate) abolished Lp(a)-mediated potentiation (Figure [Bi] and [Bii]); this was confirmed with the use of CD36^−/−^ murine platelets (Figure [Biii]).^4^ CD36-dependent platelet activation requires Src-family kinases (SFKs), and consistent with this mechanism, Lp(a)-driven potentiation of platelet aggregation was abolished in the presence of the pan SFK inhibitor PP2 (1-tert-butyl-3-(4-chlorophenyl)-1H-pyrazolo[3, 4-d]pyrimidin-4-amine) (Figure [Bii]).

**Figure.**
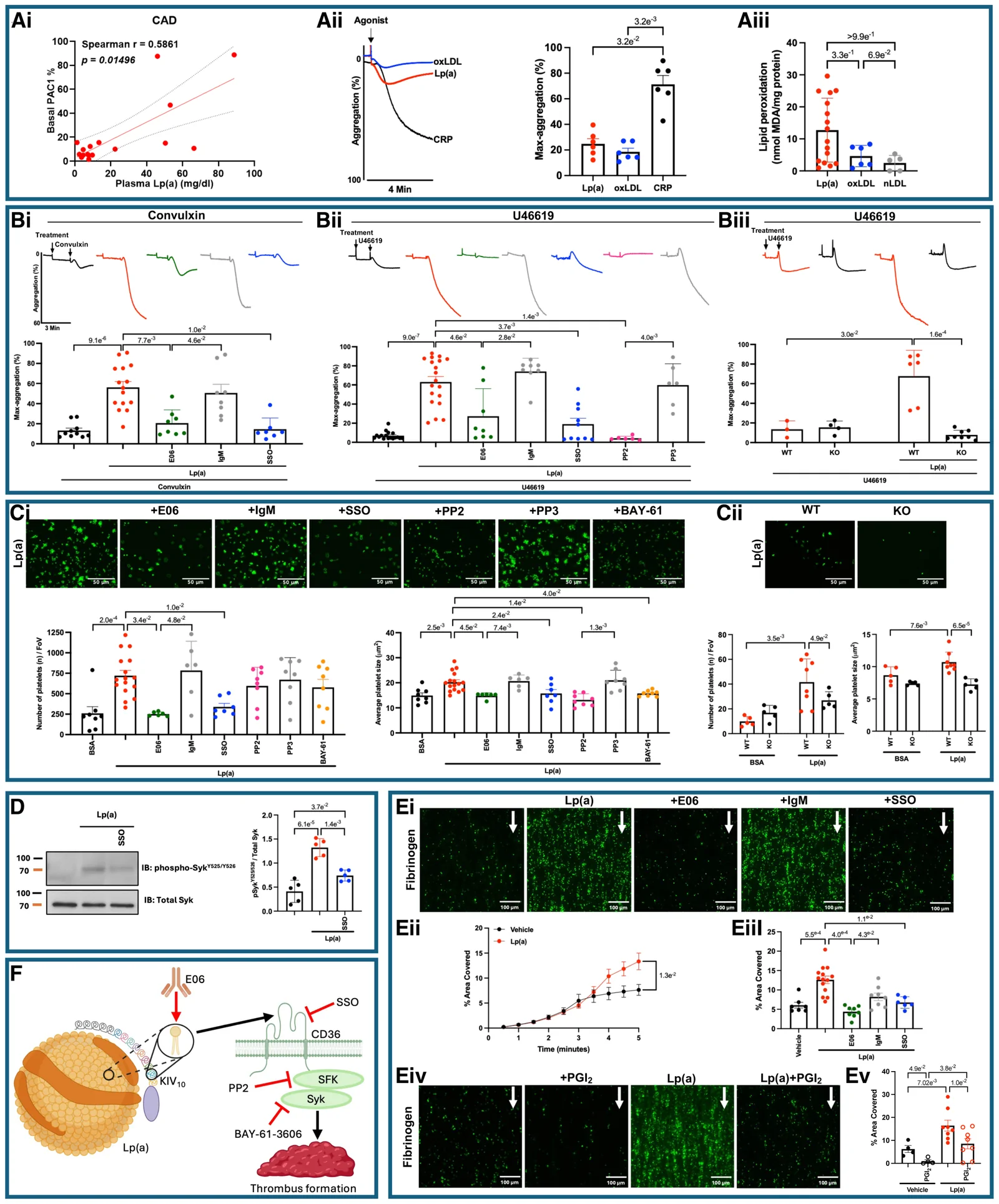
**Lipoprotein(a) (Lp[a]) drives platelet activation through oxidized phospholipids (oxPLs)–CD36 (cluster of differentiation 36). Ai**, Correlative analysis of plasma Lp(a) concentrations and basal platelet activation (%PAC-1+) in patients with coronary artery disease (CAD; n=17). **Aii**, Aggregation of human platelets (2.5×10^8^ platelets/mL) treated with Lp(a) (100 μg/mL), oxidized low-density lipoprotein (oxLDL; 100 μg/mL) or collagen-related peptide (CRP; 10 μg/mL; 4-minute, 1000 rpm; n=6); **left**, representative traces; **right**, maximum aggregation. **Aiii**, Lipid peroxidation of isolated Lp(a) (n=16), native (n=5) and oxLDL (n=6), normalized to protein concentration (excitation/emission=532/553; nmol MDA/mg protein). **B**, Aggregation of human platelets (2.5×10^8^ platelets/mL; **Bi**, n=10; **Bii**, n=17), pretreated with SSO (sulfo-N-succinimidyl oleate) (1 μM; **Bi**, n=7; **Bii**, n=10) or PP2/PP3 (20 μM, 20-minute; **Bii**, n=6), followed by Lp(a) (100 μg/mL;1-minute, 1000 rpm; **Bi**, n=15; **Bii**, n=19) and then convulxin (12.5 ng/mL; **Bi**) or U46619 (0.1 μM; **Bii**) stimulation. In some cases, Lp(a) was coincubated±E06/IgM isotype control (1 hour, 4 °C; 10 μg/mL; **Bi**, n=8; **Bii**, n=8). **Biii**, Aggregation of CD36^–/–^ (knockout [KO], n=4) or wild type (WT, n=3; C57BL/6J, 12–20 weeks, male+female) murine platelets induced by Lp(a) and U46619 (1 μM; WT, n=6, KO, n=8; **upper**, representative traces; **lower**, maximum aggregation. **C**, Adhesion and spreading of human platelets (1×10^8^ platelets/mL; n=8), pretreated with SSO (1 μM; n=7), PP2/PP3 (20 μM; n=8) or BAY-61-3606 (10 μM, 20 minutes; n=8) on Lp(a)-coated (50 μg/mL; n=16) wells, pretreated±E06/IgM (5 μg/mL, 1 hour; n=6). **Cii**, CD36^–/–^ (KO; n=5) or WT murine platelets (n=5) on Lp(a)-coated (50 μg/mL; WT, n=8; KO, n=5) wells; **upper**, representative images; **lower left**, average number of platelets adhered across 5 images (Field of View=291.23×229.82 μM); **lower right**, average platelet size (μm^2^). **D**, Washed human platelets (2.5×10^8^ platelets/mL), pretreated with SSO (1μM) and Lp(a)-stimulated (100 μg/mL, 1 minute) before immunoblotting (IB) for phospho-Syk^Y525/Y526^ and total Syk; **left**, representative blots; **right**, densitometric analysis (n=5). **E**, Human washed platelets (4×10^8^ platelets/mL; **Eii** and **Eiii**, n=7; **E**v, n=5), pretreated with SSO (1 μM;n=6) or prostacyclin (prostaglandin I_2_ [PGI_2_]; 50nM, 2 minutes; vehicle, n=4; Lp(a)-stimulated, n=8), were stimulated with Lp(a) (100 μg/mL; 37 °C, 15 minutes; **Eiii**, n=14; **Ev**, n=8) ±E06/IgM (5 μg/mL; n=8), before reconstitution with red blood cells (1:1) and perfused over fibrinogen-coated Ibidi chips (1000s^−1^, 2 minutes, 1 mg/mL). **Ei** and **Eiv**, Representative images; **Eii**, **Eiii**, and **Ev**, Percentage area covered. In all experiments, platelet donor preparations were treated with multiple independent Lp(a) preparations. Representative images reflect values close to the group median. **F**, Schematic demonstrating Lp(a)-derived oxPLs binding platelet-CD36, driving downstream signaling, including Src/Syk activation and thrombus formation (KIV_10_ [Kringle IV type 10]). Normality was assessed using the Shapiro-Wilk test, and analyses were performed using the Kruskal-Wallis test with Dunn multiple comparisons (**Aii**, **Aiii**, **B** through **D**, **Ei**, and **Eiii** through **Ev**) within each analysis, with adjusted *P* values reported; **Aii**, Spearman correlation; **Eii**, Mann-Whitney *U* test; no across-test correction for multiple testing was applied (means±SD; compared with Lp(a)-treated or control; GraphPad Prism 11.0.0). nLDL indicates native low-density lipoprotein; PAC-1, platelet activation 1; PP2, 1-tert-butyl-3-(4-chlorophenyl)-1H-pyrazolo[3, 4-d]pyrimidin-4-amine; SFK, Src-family kinase; and Syk, spleen tyrosine kinase. Created in BioRender. Naseem, K. (2026) https://BioRender.com/gerqzf5.

Beyond its soluble effects, Lp(a) binds extracellular matrix proteins within atherosclerotic plaques and may serve as an immobilized ligand for platelet capture after plaque rupture.^1^ Reflecting this, surface-immobilized Lp(a) (50 μg/mL) supported platelet adhesion and drove morphological changes, including cytoskeletal rearrangement and spreading, which were significantly diminished when Lp(a) was treated with E06 (Figure [Ci]). Furthermore, pharmacological inhibition of CD36 on human platelets with SSO (Figure [Ci]) or genetic deletion in CD36^−/−^ mice (Figure [Cii]) prevented Lp(a)-supported platelet adhesion and spreading. Concurrently, treatment with PP2 and the highly selective Syk (spleen tyrosine kinase) inhibitor BAY-61-3606 reduced platelet spreading on immobilized Lp(a) (Figure [Ci]). Supporting the functional studies, we found increased phosphorylation of Syk at activating tyrosine phospho-sites in response to Lp(a), which was attenuated by SSO (Figure [D]). These results provide evidence that Lp(a)-driven platelet activation operates through associated oxPL moieties coupled to CD36 and downstream Src/Syk signaling.

We next assessed the pathophysiological relevance of Lp(a)-mediated platelet activation under hemodynamic arterial flow. To isolate the direct effects of Lp(a) on human platelets, without interference from endogenous circulating Lp(a), we used a reconstituted blood model.^4^ Perfusion of reconstituted whole blood, pretreated with Lp(a) (50 μg/mL), over fibrinogen (1 mg/mL) promoted platelet microaggregate formation and significantly increased surface coverage compared with untreated platelets (6.1±2.0% versus 12.6±3.6%; *P*=5.5×10^−4^; Figure [Ei] through [Eiii]). Furthermore, the ability of Lp(a) to promote in vitro thrombus formation on fibrinogen was ablated by E06 and SSO (Figure [Eiii]), with surface coverage reduced from 12.6±3.6% to 4.4±1.5% (*P*=4.0×10^−4^) and 6.8±1.5% (*P*=1.1×10^−2^), respectively. Interestingly, Lp(a) diminished the antithrombotic effects of prostacyclin, a potent endogenous platelet inhibitor. Prostacyclin abolished platelet accrual on fibrinogen (0.7±1.3%; *P*=4.9×10^−2^) but was markedly less effective in the presence of Lp(a) (8.5±6.2%; *P*=3.8×10^−2^ versus prostacyclin; *P*=1.0×10^−2^ versus Lp[a] alone; *P*=5.1×10^−2^ versus untreated; Figure [Eiv] and [Ev]). Thus, oxidized Lp(a) has the capability to both promote platelet-driven thrombosis and disrupt endogenous protection afforded by the endothelium-derived prostanoid, creating a “double hit” effect on platelets that favors thrombus formation.

In summary, we demonstrate that Lp(a) promotes platelet aggregation and adhesion and enhances thrombosis under arterial flow. These findings implicate Lp(a) as a pathological mediator that may accelerate thrombosis at sites of atherosclerotic plaque rupture, where platelets encounter matrix-bound oxidized Lp(a) together with multiple physiological agonists. To our knowledge, this study provides the first direct evidence that Lp(a)-mediated platelet hyperreactivity operates through an oxPL-CD36–dependent mechanism (Figure [F]), establishing a novel mechanistic link between elevated Lp(a) and atherothrombosis. It is important to note that owing to well-established limitations in large-scale Lp(a) isolation, all experiments were standardized to protein concentration. An ELISA-based assay measured the effective Lp(a) concentrations used as low physiological concentrations, which ranged from 0.5 to 2 mg/dL, relative to the circulating human range of 1 to >200 mg/dL. The potent biological effects observed at these concentrations therefore underscore the thromboinflammatory potency of the oxPL-associated fraction of Lp(a). The central role of the prothrombotic receptor CD36^5^ and the ability of E06 to block the effects of Lp(a) strongly implicate phosphocholine-containing oxPL,^1^ in the prothrombotic effects of Lp(a). Identifying the specific oxPL species and understanding the role of apoA isoform heterogeneity in promoting platelet hyperactivity will require further detailed investigation. Together with clinical evidence that patients with markedly elevated Lp(a) derive greater benefit from prolonged dual antiplatelet therapy, our findings provide a mechanistic foundation for evaluating and refining antiplatelet strategies in patients with elevated oxLp(a) and oxPL burden.

## Article Information

### Acknowledgments

The authors thank the Bioimaging and Flow Cytometry facility staff at the University of Leeds for their support. Schematics were created with BioRender.com.

### Data Availability

The data that support the findings of this study are available from the University of Leeds on reasonable request. All data points underlying the results are presented and available as part of the article. Approval for collecting blood from healthy volunteers was granted by the School of Medicine Research Ethics Committee (SoMREC 23-001, University of Leeds). Patients were recruited under the Health Research Authority approval (24/YH/0063). All samples were obtained in accordance with the Declaration of Helsinki. All animal studies were approved by the UK Home Office (PP9539458).

### Author Contributions

E. Horner designed the research, performed the experiments, analyzed the data, and wrote the manuscript; L.T. Cheah performed the experiments, analyzed the data, and critically reviewed and edited the manuscript; T. Palmer-Dench and J.S. Khalil performed the experiments and analyzed the data; M. McKay performed the experiments; F.L. Macrae was responsible for funding acquisition; M.T. Kearney and A.J. Unsworth designed the research and critically reviewed and edited the manuscript; and K.M. Naseem was responsible for funding acquisition and project administration, designed the research, and wrote the manuscript. All authors have read and agreed to the published version of the manuscript.

### Disclosures

None.

### Supplemental Material

Major Resources Table

ARRIVE Guidelines

Uncropped Gel Blots

## Supplementary Material


