# Supplementary material for: Lipoprotein(a) Drives Platelet Hyperactivity Through Oxidized Phospholipids-CD36
Source: Circ Res. 2026 Sep 21;139(10):e329174. doi: 10.1161/CIRCRESAHA.126.329174 (PMC13686627; doi:10.1161/CIRCRESAHA.126.329174)
Supplement: Supplementary file 1 [file res-139-e329174-s001.pdf]

Major Resources Table

Mouse Models Used in This Study

Strain	Vendor or Source	Background Strain	Sex	Other Information (e.g. breeding scheme for F1, F2…if applicable)	Persistent ID / URL
C57BL/6	Charles River (Strain#:632)	C57BL/6J	M&F	This strain was purchased for breeding of wild-type colony.	https://www.criver.com/products-services/find-model/jax-c57bl6j-mice?region=3671
B6.129S1-*Cd36*^{tm1Mfe}/J	JAX (Strain #:019006)	C57BL/6J	M&F	This strain was purchased for breeding of CD36 global deficient mouse.	https://www.jax.org/strain/019006

Antibodies

Target antigen	Vendor or Source	Catalog #	Working concentration	Lot # (preferred but not required)	Persistent ID / URL
Anti-oxidised phospholipid antibody, clone E06	Sigma-Aldrich	MABS2278	100μg/ml	4389592	https://www.sigmaaldrich.com/GB/en/product/mm/mabs2278
IgM kappa, from murine myeloma	Sigma-Aldrich	M3795	100μg/ml	0000336893	https://www.sigmaaldrich.com/GB/en/product/sigma/m3795
Anti human CD41/CD61 (PAC1 clone)	BD Biosciences/ Waters Biosciences	340507	2.5μg/ml	5262559	https://www.bdbiosciences.com/en-gb/products/reagents/flow-cytometry-reagents/clinical-discovery-research/single-color-antibodies-ruo-gmp/fitc-mouse-anti-human-pac-1.340507?tab=product_details
Alexa-Flour 488 Phalloidin	Invitrogen	A12379	6.6nM	3184335	https://www.thermofisher.com/order/catalog/product/A12379
Phospho-Syk y525/526	Cell Signalling	C87C1	0.09μg/ml	25	https://www.cellsignal.com/products/primary-antibodies/phospho-syk-tyr525-526-c87c1-rabbit-monoclonal-antibody/2710
Total Syk (4D10)	Santa Cruz	SC-1240	0.2μg/ml	B1711	https://www.scbt.com/p/syk-antibody-4d10

Other

Description	Source	Persistent ID / URL
BAY-61-3606	Cayman Chemicals	https://www.biomol.com/products/chemicals/biochemicals/bay-61-3606-hydrochloride-cay11423-1
BSA	Sigma-Aldrich	https://www.sigmaaldrich.com/GB/en/product/sigma/a4503?srsltid=AfmBOooDE31jQh5sRhCuGKpP1a20b-U0eNzT8P-TPJfys5OF-kdtvH6Z
Convulxin		https://www.caymanchem.com/product/19082/convulxin?srsltid=AfmBOopDfK5NheVLjzowL3T5rru8y8lu2tj0hwKP8muMQoNVBw33t8ZC

CRP-A	PPlus	https://www.pplusmedical.com/wp-content/uploads/2026/05/CRP-A-Datasheet-Rev-10.00.pdf
Lipid Peroxidation Assay Kit	Abcam	https://www.abcam.com/en-us/products/assay-kits/lipid-peroxidation-mda-assay-kit-colorimetric-fluorometric-ab118970
Human Lipoprotein(a) ELISA Kit	Abcam	https://www.abcam.com/en-us/products/elisa-kits/human-lipoprotein-a-elisa-kit-ab212165
PGI2	Cayman Chemicals	https://www.caymanchem.com/product/18220/prostaglandin-i2-sodium-salt?srsltid=AfmBOoplzqvi5GWIqcBJMVU5rqkcMagSACDoiWkn1M9DVUs1LG2aela0
PP2	Abcam	https://www.abcam.com/en-us/products/biochemicals/pp2-src-and-rip2-kinase-inhibitor-ab120308
PP3	Abcam	https://www.abcam.com/en-us/products/biochemicals/pp3-negative-control-for-pp2-ab120617
SSO	Santa Cruz	https://www.scbt.com/p/sulfosuccinimidyl-oleate-sodium-135661-44-8?srsltid=AfmBOoptWMeReef2WN_LrkLT1_-0Nf_9CovaPOJqB24926stFdnEUx2G
U46619	Cayman Chemicals	https://www.caymanchem.com/product/16450/u-46619?srsltid=AfmBOoq5oSBLKSHxUTZmYBFtKAX2pi2x90FQRIcC5CLEw4aC7rl6UlIa

ARRIVE GUIDELINES

The ARRIVE guidelines (https://arriveguidelines.org/) are a checklist of recommendations to improve the reporting of research involving animals. Key elements of the study design should be included below to better enable readers to scrutinize the research adequately, evaluate its methodological rigor, and reproduce the methods or findings.

Study Design

Groups	Sex	Age	Number (prior to experiment)	Number (after termination)	Littermates (Yes/No)	Other description
Group 1 (Control)	M&F	12-20weeks	8	0	No	
Group 2	M&F	12-20weeks	8	0	No	

Sample Size

Sample sizes for murine experiments were determined based on previous experience with the experimental models and assays, previously observed biological variability alongside feasibility considerations. Group sizes were selected in accordance to animal-use considerations, whilst also providing sufficient biological replicates to detect biologically meaningful differences between experimental groups. Final group sizes varied between experiments because of experimental avalibility.

Inclusion and Exclusion Criteria

No criteria was used for including or excluding animals during the experiment or data points during the analysis.

Randomization

Following genotyping, CD36 deficient and WT control mice were allocated to study groups and animal identities coded by an independent researcher.

Blinding

Animal identities were coded such that investigators performing experiments, outcome assessments, and data analyses were blinded to genotype until completion of the study.
