# Supplementary material for: Lipoprotein(a) Drives Platelet Hyperactivity Through Oxidized Phospholipids-CD36
Source: Circ Res. 2026 Sep 21;139(10):e329174. doi: 10.1161/CIRCRESAHA.126.329174 (PMC13686627; doi:10.1161/CIRCRESAHA.126.329174)
Supplement: Supplementary file 2 [file res-139-e329174-s002.pdf]

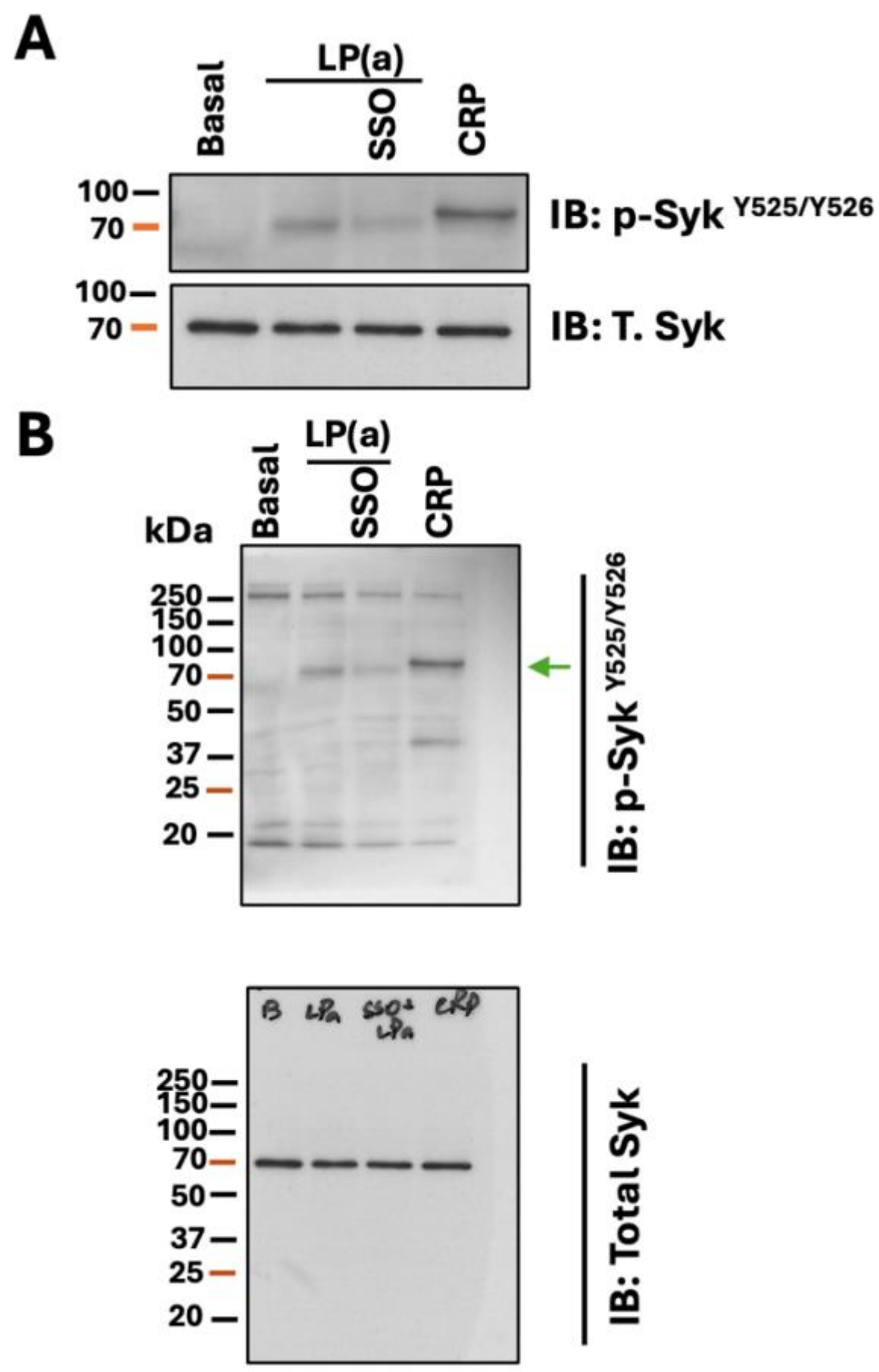

(A) Representative image for Figure D. (B) **Full uncropped blots** for: top, phospho-Syk$^{Y525/526}$; bottom, total Syk for platelet donor 1.

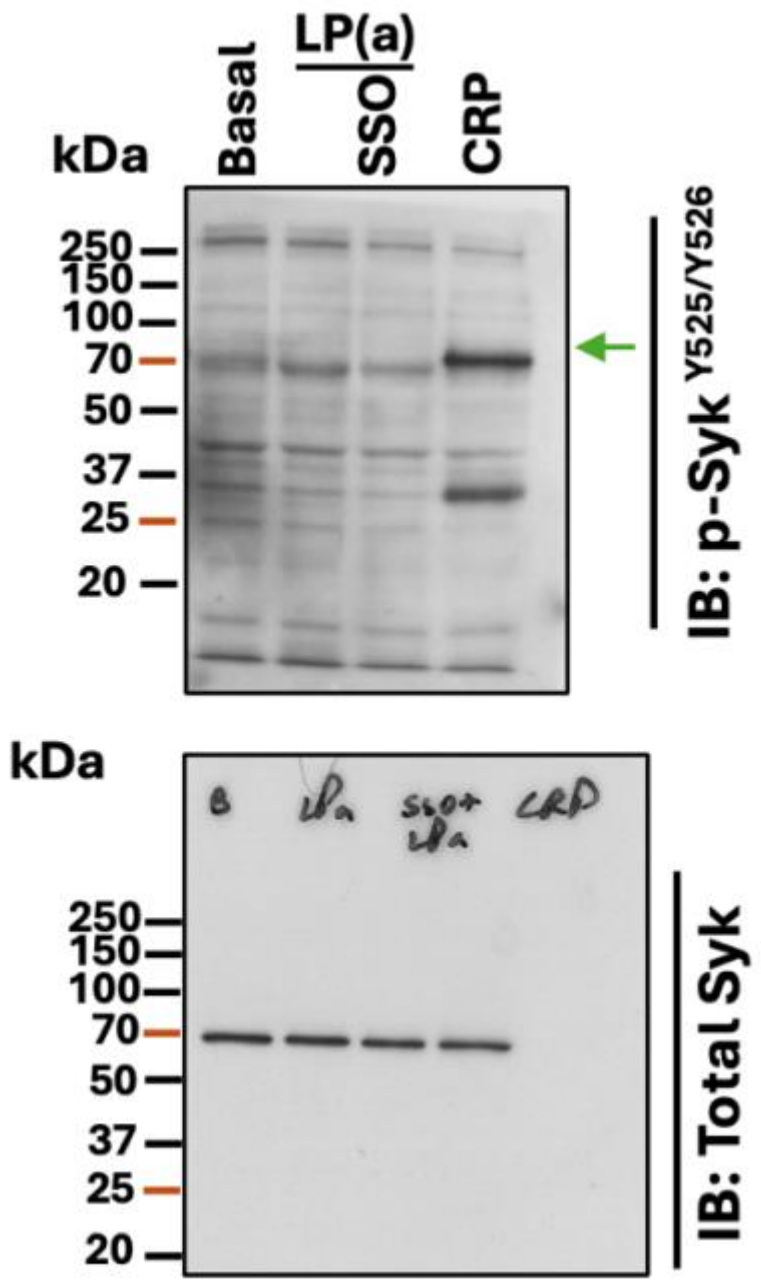

Full uncropped blots for: top, phospho-Syk$^{Y525/526}$; bottom, total Syk for platelet donor 2.

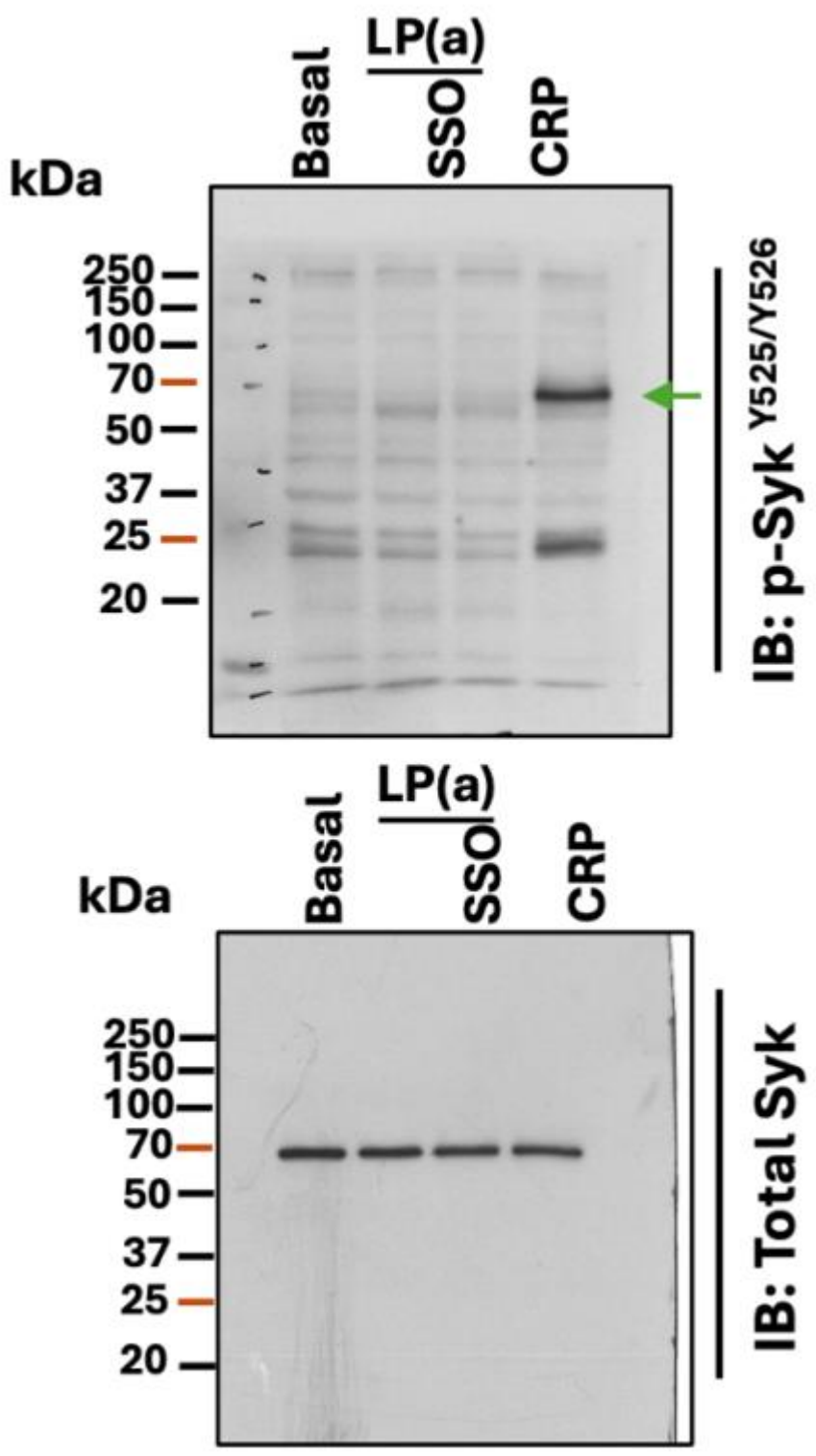

Full uncropped blots for: top, phospho-Syk$^{Y525/526}$; bottom, total Syk for platelet donor 3.

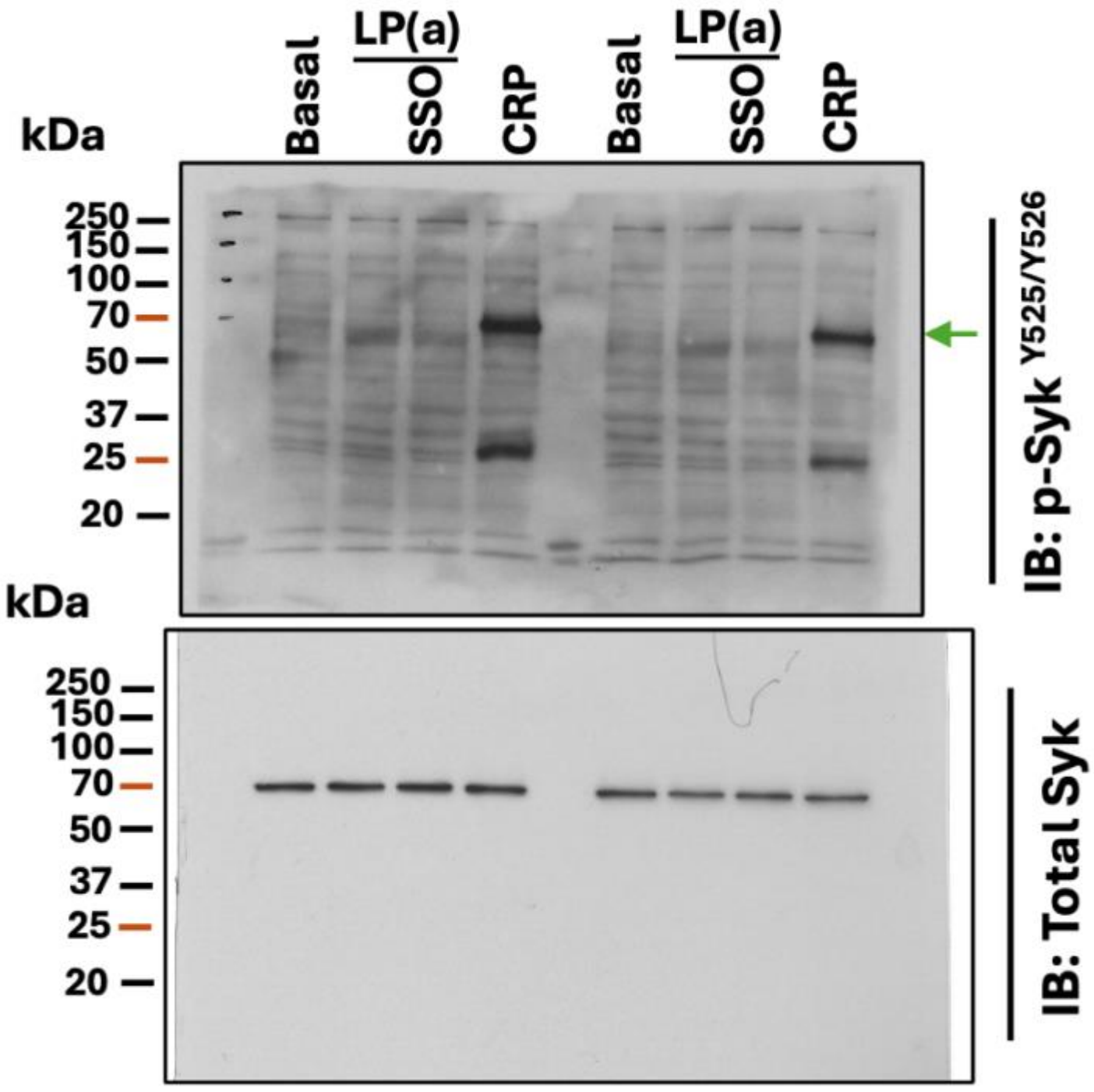

Full uncropped blots for: top, phospho-Syk$^{Y525/526}$; bottom, total Syk for platelet donor 4 and 5.
